# Pulmonary sarcomatoid carcinoma: University of Cincinnati experience

**DOI:** 10.18632/oncotarget.23468

**Published:** 2017-12-18

**Authors:** Nagla Abdel Karim, James Schuster, Ihab Eldessouki, Ola Gaber, Tariq Namad, Jiang Wang, Changchun Xie, John C. Morris

**Affiliations:** ^1^ Vontz Center for Molecular Studies, University of Cincinnati, Cincinnati, OH 45219, USA; ^2^ University of Cincinnati College of Medicine, Cincinnati, OH 45267, USA; ^3^ Military Hospital of Mohammed V, Department of Medical Oncology, Quartier Irfane, Rabat 10080, Morocco; ^4^ UC Health University Hospital, Department of Pathology and Laboratory Medicine, Cincinnati, OH 45219, USA; ^5^ Division of Biostatistics and Bioinformatics, Department of Environmental Health, Cincinnati, OH 45267, USA

**Keywords:** chemotherapy, lung cancer, non-small cell lung cancer, sarcomatoid

## Abstract

**Objectives:**

To review the outcomes of treatment in patients with pulmonary sarcomatoid carcinoma (PSC) treated at the University of Cincinnati Medical Center (UCMC).

**Results:**

There was no significant difference in survival of patients treated with chemotherapy alone (median, 256 days) compared to patients not undergoing treatment (median, 205.5 days). Patients who underwent surgery and adjuvant chemotherapy showed a trend in improvement of survival (median, 457.6 days). Patients requiring only surgery had the longest OS of 713.5 days.

**Conclusions:**

Systemic chemotherapy alone did not improve survival in patients with PSC. Surgery provides the greatest overall survival benefit and adjuvant chemotherapy may also improve survival.

**Methods:**

From 2000 to 2014, twenty-five patients with pathologically confirmed PSC were treated at UCMC. The outcomes were retrospectively analyzed by treatment with overall survival (OS) as the endpoint.

## INTRODUCTION

Pulmonary sarcomatoid carcinoma (PSC) is a rare subtype of poorly differentiated non-small-cell lung carcinoma (NSCLC) that accounts for 0.1%–0.4% of all lung cancers [1–3]. PSC carries a poor prognosis compared to the other types of NSCLC even in early stage disease [4]. PSC typically occurs in older, heavy smoking men and has a predilection for upper lobe involvement [5, 6]. The sensitivity PSC to chemotherapy is controversial; however, surgery in early stage disease has demonstrated survival benefit [5, 7].

Pathologically PSC demonstrates both epithelial and mesenchymal elements in the tumor distinguishing it from other types of NSCLC. PSC was initially described Virchow in 1865 as a “biphasic” lesion of adenocarcinomatous or squamous cell components along with spindle or giant cell elements forming at least 10% of the tumor mass [8]. This description fulfills the current WHO criteria for the diagnosis of PSC. Based on the WHO criteria, five subgroups of PSC exist: pleomorphic carcinoma, spindle cell carcinoma, giant cell carcinoma, carcinosarcoma, and pulmonary blastoma [3]. Overall, it has proven difficult to formulate treatment recommendations for PSC due to its rarity, rapid progression and short survival, and heterogeneous pathological qualities.

Genomic analysis has revealed a common origin of both the epithelial and mesenchymal elements and it is thought that epithelial-mesenchymal transition (EMT) is the mechanism that gives rise to this tumor, with the epithelial elements, adenocarcinoma or squamous components, having undergone a transition to a poorly-differentiated mesenchymal or “sarcomatoid” phenotype with the expression of mesenchymal proteins such as vimentin [9, 10]. Ultimately, efforts to study PSC have been hindered by the rarity of this NSCLC variant.

## RESULTS

We identified 25 patients with a diagnosis of PSC (Table 1). The 16 men and 9 women had a median age of 57 years (range, 31–83 years). Chemotherapy was most often used in advanced stage disease (stage 3, 4). Unfortunately, treatment with systemic chemotherapy did not show a significant improvement in outcome (*p =* 0.451, HR 0.638). Patients who underwent surgery and systemic chemotherapy showed a trend toward improvement in outcome (*p =* 0.08 and HR 0.04). Their median OS was 457.6 days (95% CI 206–1187 days). Patients who underwent surgical resection only had the best median overall survival of 713.5 days (95% CI, 246–1138 days) (Table 2).

**Table 1:** Baseline characters and demographics of sarcomatoid lung cancer patients (*n* = 25)

Patient	Age (years)	Gender	Race	Smoking, (PPY)
**Pt_1**	57	M	AA	Smoker, 40
**Pt_2**	56	M	W	Smoker, 40
**Pt_3**	56	F	W	Non-smoker
**Pt_4**	31	F	W	Smoker, 43
**Pt_5**	76	M	W	Smoker, 43
**Pt_6**	51	M	W	Smoker, 60
**Pt_7**	73	F	W	Smoker, 54
**Pt_8**	83	M	W	Smoker, 70
**Pt_9**	62	M	W	Smoker, 40
**Pt_10**	47	M	W	Smoker, 20
**Pt_11**	51	M	AA	Smoker, 50
**Pt_12**	68	F	W	Smoker, 50
**Pt_13**	69	M	W	Non-smoker
**Pt_14**	66	M	W	Smoker, 56
**Pt_15**	68	M	W	Smoker, 45
**Pt_16**	57	M	W	Smoker, 40
**Pt_17**	67	F	W	Smoker, 50
**Pt_18**	54	M	AA	Smoker, 38
**Pt_19**	84	F	W	Non-smoker
**Pt_20**	57	F	W	Smoker, 36
**Pt_21**	71	M	W	Smoker, 43
**Pt_22**	54	M	W	Non-smoker
**Pt_23**	70	M	W	Smoker, 56
**Pt_24**	59	F	AA	Smoker, 40
**Pt_25**	59	F	W	Smoker, 50

Abbreviations: EGFR, epidermal growth factor receptor;

EML-4/ALK, echinoderm-like microtubule-like protein-4/anaplastic lymphoma kinase; HR, hazard ratio;

NSCLC, non-small cell lung cancer;

PSC, pulmonary sarcomatoid carcinoma;

OS, overall survival,

M: male, F: female, W: white American, AA: African American,

PPY: Pack per year.

Pt: patient.

**Table 2:** Treatment strategies adopted in the sarcomatoid lung cancer patients according to their stage (*n* = 25)

Patient	Stage	Surgery	Radiotherapy	Chemotherapy
**Pt_1**	Stage III	No	Yes; concurrent chemoradiation	Neoadjuvant; Cisplatine-Gemzar, carbo-taxol
**Pt_2**	Stage III	No	Yes; concurrent chemoradiation	1st L Carbo Alimta + Radiation, 2d L: Taxoter (4C)àDP, 3th L:Gemzar (3C)àDP, 4th L: Phase 1 study
**Pt_3**	Stage II	Right middle lobectomy	Left L2 pedicle (24 Gy)	4 cycles Cisplatin-gemzar
**Pt_4**	Stage Ib	RLL lobectomy	No	No
**Pt_5**	Stage Ib	Right VTAS + RLL lobectomy	No	No
**Pt_6**	Stage II	Right thoracotomy with upper lobe sleeve lobectomy	No	No
**Pt_7**	Stage III	Sub carinal lymph node EBUS aspiration	No	Carboplatine + alimta
**Pt_8**	Stage IV	No	No	No
**Pt_9**	Stage IIb	Left thoracotomy + LLLlobectomy + mediastinal lymph nodes resection	Adrenal radiation	Adjuvant Carboplatine-Taxol (6C) àDP
**Pt_10**	Stage IV	No	Right lung +mediastinum (60 Gy)	No
**Pt_11**	Stage IV	No	No	No
**Pt_12**	Stage II	Right posterior upper lobectomy + mediastinal lymph node resection	No	No
**Pt_13**	Stage Ib	RLL VATS lobectomy	No	No
**Pt_14**	Stage Ib	Right VTAS RLL lobectomy	No	No
**Pt_15**	Stage II	Right parietal resection	No	No
**Pt_16**	Stage III	No	No	Carboplatine + Taxol (1 cycle)
**Pt_17**	Stage II	Left posterior thoracotomy with wedge resection	No	No
**Pt_18**	Diagnosed on Autopsy	No	No	No
**Pt_19**	Stage II	RUL lung lobectomy + thoracic Lymph nodes adenectomy	No	No
**Pt_20**	Stage II	Left thoracotomy + left upper lobectomy	No	No
**Pt_21**	Stage Ib	RUL lobectomy	Lower back palliative radiation	No
**Pt_22**	Stage IV	No	No	No
**Pt_23**	Stage IV	Brain surgery for lung cancer metastasis	Brain radiation	No
**Pt_24**	Stage II	Right Lung mass resection Brain metastasis resection	Local chest radiation	No
**Pt_25**	Stage III	No	No	Carboplatine-Taxol

**Figure 1:**
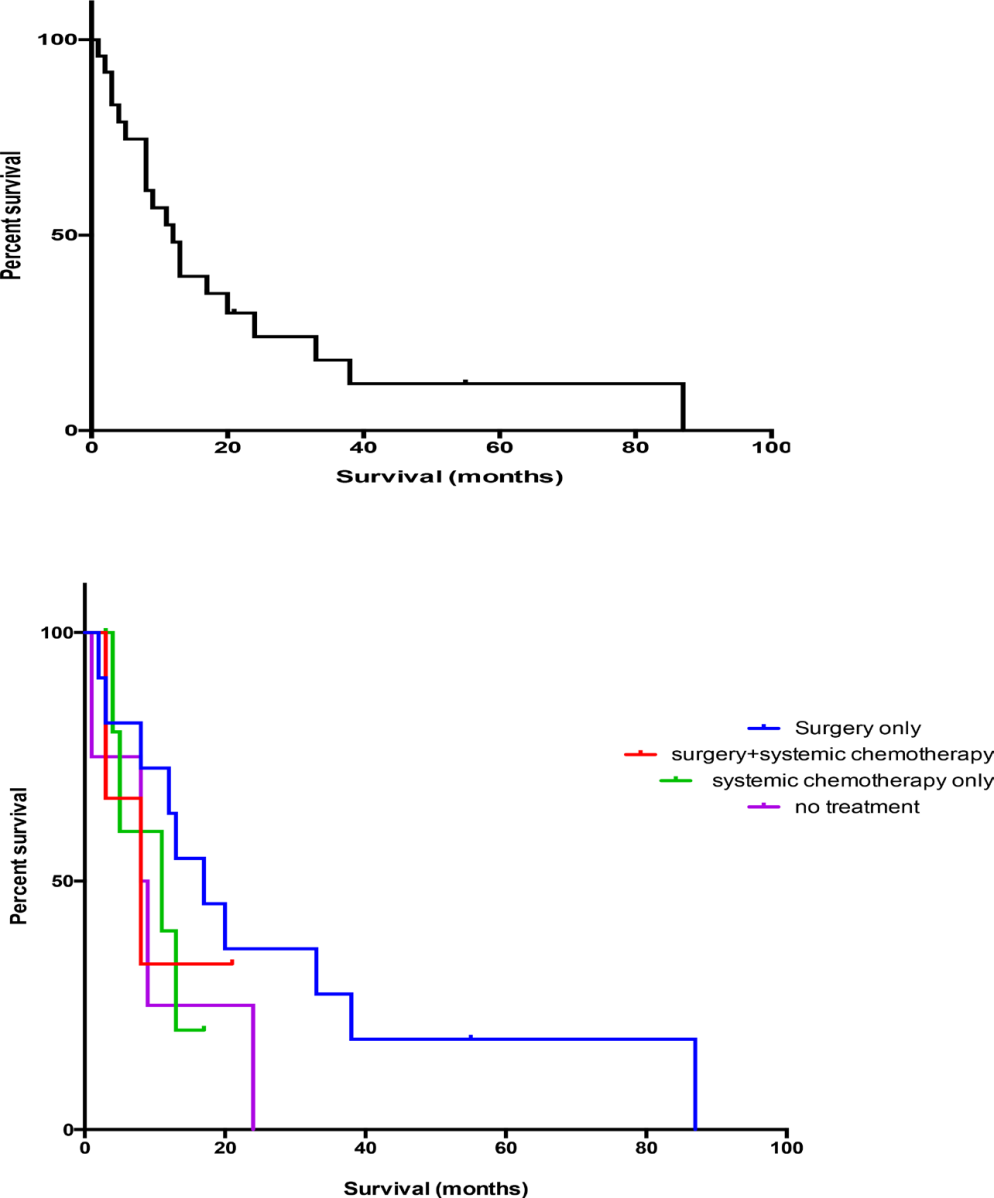
Survival of sarcomatoid lung cancer patients (*n* = 25)

All patients treated with systemic chemotherapy had a median OS of 375 days (95% CI, 114–600 days). In patients treated with systemic chemotherapy only, their median OS was 256 days (95% CI 114–600 days). These were patients with advanced disease at diagnosis. Patients who received neither chemotherapy nor surgery had a median OS of 205.5 (95% CI 98–447 days), (Figure 1).

**Figure 2:**
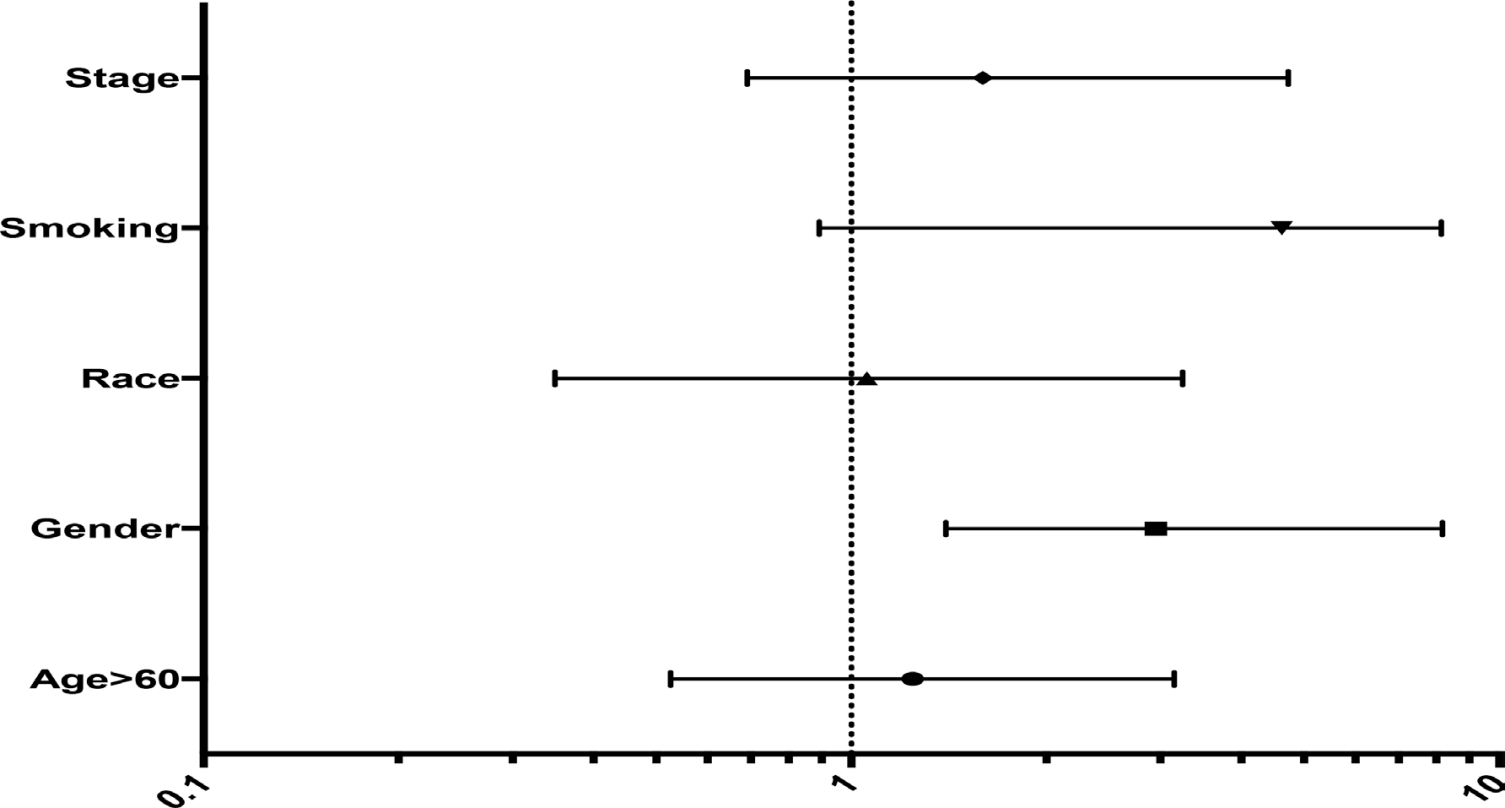
Forest Plot showing hazard ratio for different parameters Smoking had the highest impact followed by stage, being the main detrimental factor for the type treatment the patient receives.

Age (*p* = 0.39 and HR 0.98) showed a trend for overall survival, on contrary to gender where female showed better OS response. The patient who demonstrated the longest OS received systemic therapy including cisplatin, gemcitabine, docetaxel and crizotinib. Two of the patients in the study demonstrated EML-4/ALK translocations (Figure 2).

## DISCUSSION

The rarity pulmonary sarcomatoid carcinoma and the difficulty of pathological diagnosis make it a difficult malignancy to study. A search of PubMed entries between 01/01/1995 and 09/01/2016 using the keywords “pulmonary”, “sarcomatoid”, and “carcinoma” identified 11 retrospective studies addressing chemotherapy and surgical resection of PSC. Two studies have reported the median overall survival of PSC treated with systemic chemotherapy compared to no chemotherapy. Although limited in size, our study quantifies the median overall survival of patients with PSC treated with systemic chemotherapy and surgery, surgery alone, systemic chemotherapy alone, and no treatment. No study to date has compared the outcomes of these different patient groups in a single study. Table 3 shows our findings compared to other reports.

**Table 3:** Median overall survival in patients with pulmonary sarcomatoid carcinoma (PSC) with various treatments

Location/Date of Study	Patients	Systemic Chemotherapy and Surgery	Surgery Only	Systemic Chemotherapy Only	Without Systemic Chemotherapy or Surgery	Reference
UCMC, 2016	21	457.5	713.5	256	205.5	-
China, 2013	51	516.8	167.2	-	106.8	2
France, 2016	93	-	-	130.7	-	1
Japan, 2015	16	-	-	212.8*	-	15
Italy, 2003	75	-	577.6	-	-	29

*Median overall survival of patients with only the pleomorphic subtype of pulmonary sarcomatoid carcinoma.

In studies that measure median overall survival in months, 1 month = 30.4 days.

Prognosis of PSC is poor, even when compared to other types of NSCLC. The efficacy of systemic chemotherapy has varied with some studies showing no overall survival benefit and others showing a modest benefit [2, 5, 6, 11]. Our study demonstrates advanced PSC has minimal sensitivity to chemotherapy, with an increase median OS of 256 days with systemic chemotherapy only compared to 205.5 days without any treatment. Chemotherapy does not appear to significantly improve overall survival and may not be useful in advanced disease.

Surgery in early stage operable PSC has proven to be the greatest overall survival benefit and remains the standard of care among candidates. The results from our study are consistent with other reports and demonstrate an overall survival at 713.5 days with surgical intervention. Additionally, in patients who required chemotherapy, the patients who underwent systemic chemotherapy and surgery had an OS of 457.5 days compared to 256 days with chemotherapy alone.

In non-small cell lung carcinoma, alternatives to chemotherapy are under investigation with the development of molecular targeted therapies. Unfortunately, little data is available regarding targeted therapy in PSC. Studies have shown that the prevalence of epidermal growth factor mutations (EGFR) mutation in PSC is similar to that of lung adenocarcinoma with a range of 0%–28% [12–16]. The efficacy of EGFR tyrosine kinase inhibitors in PSC has varied between studies, and the results have been indeterminate [15, 17]. Even less data is available regarding the ALK translocations. Studies have reported 3–5% of NSCLC demonstrate an EML4-ALK fusion gene [18–20]. In PSC, single cases of the EML4-ALK rearrangements have been reported and the effects of targeted therapies against this mutation have shown to be varied, but mostly ineffective or transient responses [6, 20–23].

In our study, two patients demonstrated EML4-ALK translocations. One of the patients received crizotinib and had the longest overall survival compared to the other patients studied. In addition to EGFR mutations and ALK translocations, MET amplification and MET exon 14 skipping were recently described in PSC, providing another possibility for targeted therapy [12]. Considering that crizotinib targets both ALK and MET mutations, this agent may play an important role in treating patients with PSC [24]. Additional studies are required to determine the efficacy of molecular targeted therapies against PSC.

In addition to chemotherapy and targeted therapy, immunotherapy has demonstrated encouraging results in the treatment of NSCLC. Specifically, monoclonal antibodies directed against the programmed death 1 (PD-1)/PD ligand-1 (PD-L1) system improved overall survival in NSCLC compared to chemotherapy after disease progression [25]. PD-1 and PD-L1 expression has been examined in PSC. Two studies found a prevalence of PD-L1 expression in 53% and 69.2% of sarcomatoid carcinomas, respectively [26, 27]. In a study evaluating pleomorphic carcinomas, a subtype of PSC, the prevalence was even higher at 90.2% [28]. This suggests PSC expresses greater levels of PD-L1 compared to other NSCLCs and may represent a potential therapeutic target for PSC.

## CONCLUSIONS

Overall, pulmonary sarcomatoid carcinoma is a rare, undifferentiated NSCLC histology that has a poor prognosis. Surgery remains the best option in early stage disease. Patients with PSC do not seem to benefit from systemic chemotherapy except perhaps as adjuvant treatment after surgery. Adjuvant chemotherapy does seem to improve overall survival and should be considered in treatment of PSC. Larger prospective studies are needed to further define the efficacy and role of systemic chemotherapy in patients with PSC. Other therapeutic agents, including targeted therapies and immunotherapy also require additional research.

## MATERIALS AND METHODS

This retrospective study included all patients with a pathologically confirmed diagnosis of PSC treated at the University of Cincinnati Medical Center (UCMC) between the years 2000–2014 obtained by a review of a pathology database and the institutional tumor registry for these years. Death was considered as the study endpoint. Kaplan-Meier analysis was used to calculate median overall survival (OS) and 95% confidence intervals (CI). The Cox model was used to test the chemotherapy effect adjusted for age, sex and surgery, and determine hazard ratios (HR). Data was analyzed using SAS^®^ version 9.4.
